# Support vector machine (SVM) based multiclass prediction with basic statistical analysis of plasminogen activators

**DOI:** 10.1186/1756-0500-7-63

**Published:** 2014-01-27

**Authors:** Selvaraj Muthukrishnan, Munish Puri, Christophe Lefevre

**Affiliations:** 1 Fermentation and Protein Biotechnology Laboratory, Department of Biotechnology, Punjabi University, Patiala, India, 2CSIR-IMTECH, Chandigarh, India; 2 Institute of Microbial Technology, Sector-39A, Chandigarh, India; 3 Centre for Chemistry and Biotechnology, Deakin University, Geelong, Victoria 3217, Australia

**Keywords:** Pg-activators, Plasminogen activators, Streptokinase, Staphylokinase, Urokinase, Tissue plasminogen activators, SAK, SK, UK, tPA, Comparative analysis, SVM, Support vector machine

## Abstract

**Background:**

Plasminogen (Pg), the precursor of the proteolytic and fibrinolytic enzyme of blood, is converted to the active enzyme plasmin (Pm) by different plasminogen activators (tissue plasminogen activators and urokinase), including the bacterial activators streptokinase and staphylokinase, which activate Pg to Pm and thus are used clinically for thrombolysis. The identification of Pg-activators is therefore an important step in understanding their functional mechanism and derives new therapies.

**Methods:**

In this study, different computational methods for predicting plasminogen activator peptide sequences with high accuracy were investigated, including support vector machines (SVM) based on amino acid (AC), dipeptide composition (DC), PSSM profile and Hybrid methods used to predict different Pg-activators from both prokaryotic and eukaryotic origins.

**Results:**

Overall maximum accuracy, evaluated using the five-fold cross validation technique, was 88.37%, 84.32%, 87.61%, 85.63% in 0.87, 0.83,0.86 and 0.85 MCC with amino (AC) or dipeptide composition (DC), PSSM profile and Hybrid methods respectively. Through this study, we have found that the different subfamilies of Pg-activators are quite closely correlated in terms of amino, dipeptide, PSSM and Hybrid compositions. Therefore, our prediction results show that plasminogen activators are predictable with a high accuracy from their primary sequence. Prediction performance was also cross-checked by confusion matrix and ROC (Receiver operating characteristics) analysis. A web server to facilitate the prediction of Pg-activators from primary sequence data was implemented.

**Conclusion:**

The results show that dipeptide, PSSM profile, and Hybrid based methods perform better than single amino acid composition (AC). Furthermore, we also have developed a web server, which predicts the Pg-activators and their classification (available online at http://mamsap.it.deakin.edu.au/plas_pred/home.html). Our experimental results show that our approaches are faster and achieve generally a good prediction performance.

## Background

Plasminogen activators are serine proteases which convert plasminogen to plasmin, thus promoting fibrinolysis. The identification of Pg-activators is very important due to their function in blood clot formation. The fibrinolytic system more appropriately referred as plasminogen (Pg) activator system, controls not only the intravascular fibrin deposition, but also participates in a wide variety of physiologic and pathologic processes. The Pg-activators cleave plasminogen to produce two chains of active plasmin by a single proteolytic cleavage of the Arg560-Val562 peptide bond [1]. Plasmin is responsible for the degradation of blood clots. Broadly, the Pg-activators are classified into two types based on their mechanism of function; direct and indirect activators. Pg-activators such as tissue plasminogen activator (tPA), widely found in tissues, and urokinase (UK), originally identified in urine, are classified into direct or multiple domain activators, since they have the functional capacity to convert plasminogen to active plasmin by cleaving the Arg-Val bond on the single-chain plasminogen to release two plasmin chains. UK is a 411 residue protein, consisting of three domains and is activated by proteolytic cleavage between L158 and I159 [2,3]. In contrast, Pg-activators of bacterial origins, such as Streptokinase (SK) and Staphylokinase (SAK), are often indirect activators [4-6]. Usually, indirect Pg-activators serve as co–factors of plasminogen, forming an active 1:1 stoichiometric complex with plasminogen/plasmin that degrades fibrin clots [7]. SK is a single chain polypeptide made up of 414 amino acid residue and has a molar mass of 47 kDa. SK by itself is not a plasminogen activator, but it binds with free circulating plasminogen to form a complex (1:1), which can convert additional plasminogen to plasmin. Similarly, bacterial SAK, consists of a single polypeptide chain of 136 amino acids. The SAK–plasminogen complex is however inactive, until it is converted to an active SAK-plasmin complex by other Pg-activators [8,9]. The Pg-activators of eukaryotic origin UK and tPA are trypsin like serine protease that activate plasminogen directly and are non-immunogenic. By contrast, bacterial pg-activators are generally indirect plasminogen activators which produce systemic fibrinolysis. tPA and SK are widely used thrombolytic agents for therapy. As tPA is a eukaryotic protein, it is difficult and costly to produce, but SK and SAK can be easily produced. However, it is widely accepted that these two therapeutic molecules suffer from serious complications such as immunogenicity (in SK), brain hemorrhage (SK as well tPA) and unwanted bleeding episodes. Therefore, identifying novel molecules which are really good therapeutics without complications is a desired but challenging task. This may require extensive bacterial screening or the development of other methods to search for new molecules. Nevertheless, there is a need to improve the efficacy of these Pg-activators and also a need to identify novel Pg-activators with more favorable Pg-activation characteristics.

With the escalation of prokaryotic genome sequencing, there is an ever-increasing need for prediction tools and databases to characterize and present data relating to particular gene functions. The large number of hypothetical protein sequences available demands user-friendly computational tools to facilitate various functional genomic analyses. There are many statistical, similarity and evolutionary analysis available for protein and DNA [10-12] and, various prediction databases are available [13]. However, to our knowledge, no prediction tools are currently available to predict Pg-activators from protein sequences. According to the NCBI database, 82 genome, 2003 genes and 6200 proteins have been identified as plasminogen activator related sequence sources. With the sequencing of an increasing number of genomes, a large number of novel Pg-activator may remain to be discovered. For example, beside bacterial peptides, pg-activators have been identified in snake venom and Vampire bat saliva [14,15].

Therefore, we have made a systematic attempt to develop a method for recognizing Pg-activators and their subfamilies. We have designed a method, which is able to recognize the four subfamilies of SAK, SK, tPA and UK. The classification and assignment of pg-activators to various subfamilies was done on the basis of amino (AC), dipeptide composition (DC), PSSM profile and Hybrid approach using a machine learning based support vector Machine (SVM) methodology. The SVM based predictive approach is widely used to handle large data and it has been shown to perform well in multiple areas of biological data analysis, including classification, protein functions and type identification [16]. In this study, we have implemented SVM classifiers and used five-fold cross validation to evaluate the performance of all classifiers. For this, the dataset was randomly partitioned into five equal sets and evaluated five times with each distinct set used as input and the remaining four sets used for training. Our prediction tool can directly predict Pg-activator and their classification. Apart from this prediction study, a tool should provide additional basic information on input sequences. For this, we have also implemented programs to calculate the molecular weight, amino acid residues calculation and percentage of amino acid composition. Therefore, this tool will serve not only for prediction but also to know the basic properties of input sequences.

## Results

### Amino acid composition analysis

**Figure 1:**
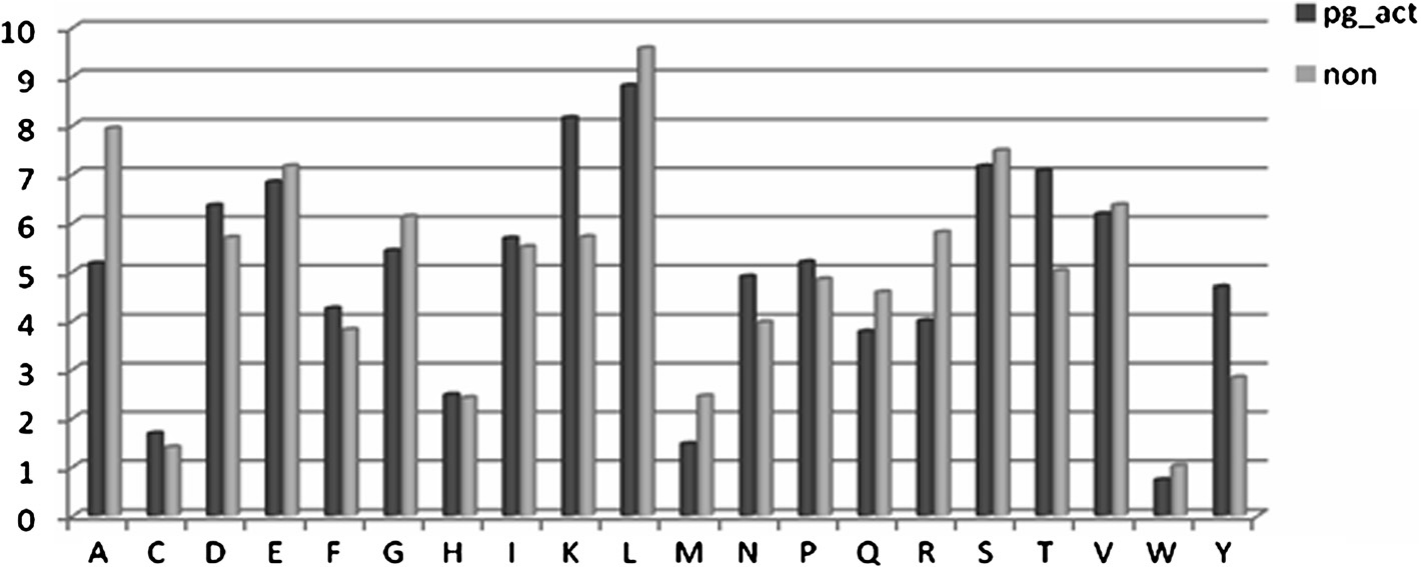
The percentage of average amino acid composition of Pg vs non Pg-activators (X-axis: amino acid residues and Y-axis: number of amino acid in percentage).

**Figure 2:**
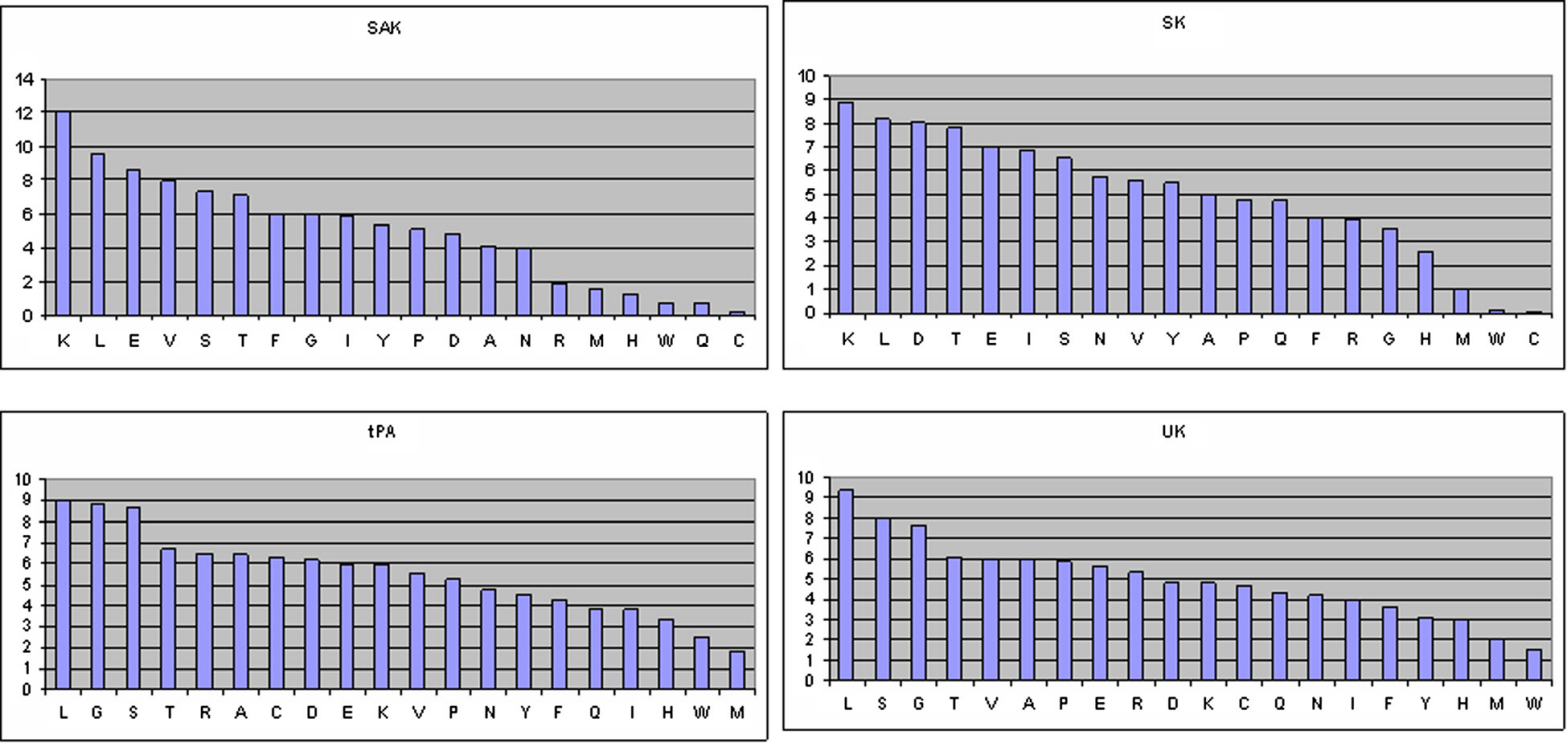
Amino acid composition comparisons of four types of Pg-activators (SAK, SK, tPA and UK), arranged as maximum to minimum number of residues (X-axis: amino acid residues and Y-axis: the number of amino acid in percentage).

When the amino acid composition of Pg-activators sequences were compared to non-Pg-activators proteins, it was observed that certain types of residues are (e.g., Lys, Trp and Tyr) are present at significantly higher frequencies in Pg-activators (Figure 1). As shown in Figure 2, Cys and Trp are less common in Pg-activators (SK and SAK) of prokaryotic origin compared to eukaryotic Pg-activators. Interestingly, Ala, Gly, Argis comparatively higher in eukayotic Pg-activators (tPA and UK).

### Amino acid composition SVM modules

Pg-activator prediction models were first developed using amino acid composition of the Pg-activators through support vector machines (SVM). SVM was trained on the different data sets using the SVM_light [17,18] implementation. First, SVM classifiers were developed using an amino acid composition vector of 20 dimensions. SVM Kernels and parameters were optimized for the best discrimination between positive and negative protein sequence data sets. Amino acid composition based prediction of Pg- activators (SK, SAK, UK and tPA) against non Pg-activators resulted in a maximum accuracy of 88.37% with 95.24%, 83.50%, 0.87 sensitivity, specificity and Mathew correlation coefficient (MCC) respectively. Finally, individual Pg-activator sub-class (SAK, SK, tPA and UK) predictions, where one sub-class of Pg- activators were considered as positive and all other sub-class activators taken as negative for SVM training [19,20] resulted in accuracy of 96.06%, 95.77%, 95.83%, 90.68% with 0.93, 0.95, 0.97, 0.93 of MCC for SAK, SK, tPA and UK subfamilies respectively (Table 1).

**Table 1:** The performance of various SVM models of Pg-activators (SAK, SK, tPA and UK) with non Pg- activators, was developed using AC, DC,PSSM profiles and Hybrid models in five-fold cross validation

	**ACC(%)**	**SN(%)**	**SP(%)**	**MCC**	**Parameters**	
***Methods***					**γ**	**C**
**AC**	88.37	95.24	83.50	0.87	25	450
**DC**	84.32	97.01	75.31	0.83	3	375
**PSSM**	87.61	95.77	81.81	0.86	3	400
**Hybrid (AC + DC)**	85.63	97.71	77.06	0.85	1	450

AC- Amino acid composition, DC dipeptide composition, PSSM position specific scoring matrix, ACC accuracy, SN- Sensitivity, SP- specificity, MCC- Mathews correlation coefficient,C: tradeoff value, γ- gamma factor (a parameter in RBF kernel).

### SVM modules using dipeptide composition

Dipeptide composition based SVM methods are generally more successful than single amino acid composition method. Here, SVM_classifiers have also been developed on dipeptide composition represented by a 400 dimension of vector (20 × 20) of dipeptide frequencies. During the optimization of kernel parameter **γ** and trade-off parameter C, better prediction performance as obtained with γ = 3 and C = 375. Based on these parameters, we have developed models to discriminate Pg-activators from non-Pg-activators sequences. As shown in Table 1, the SVM based model was able to achieve a maximum accuracy of 84.32% to 97.01%, 75.31% sensitivity, specificity and 0.83 MCC. Further classification models for sub-classification of pg-activators have been developed and resulted in accuracy of 86.82%, 86.40%, 92.70%, 87.03% of SAK, SK, tPA and UK respectively, which was shown in Table 2.

**Table 2:** The performance of various SVM models was developed using AC, DC, PSSM profiles and Hybrid methods on the individual Pg-activators SAK, SK, UK and tPA in five-fold cross validation

***Proteins***	***Methods***	***ACC(%)***	***SN(%)***	***SP(%)***	***MCC***	***Parameters***	
						**γ**	**C**
**SAK**	**AC**	96.06	92.30	96.91	0.93	3	300
	**DC**	86.82	87.08	86.76	0.83	3	75
	**PSSM**	93.98	92.28	94.34	0.92	1	300
	**Hybrid**	91.72	96.63	90.62	0.93	1	150
**SK**	**AC**	95.77	99.05	92.92	0.95	3	275
	**DC**	86.40	93.12	80.56	0.82	10	25
	**PSSM**	97.10	100	94.44	0.97	1	400
	**Hybrid**	90.75	99.05	83.55	0.90	1	250
**tPA**	**AC**	95.83	100	95.71	0.97	50	100
	**DC**	92.70	70.58	93.35	0.78	15	450
	**PSSM**	97.73	100	97.67	0.98	4	200
	**Hybrid**	92.69	75.00	93.20	0.81	10	450
**UK**	**AC**	90.68	100	86.77	0.93	3	300
	**DC**	87.03	95.03	83.66	0.87	15	500
	**PSSM**	92.06	93.19	91.56	0.90	5	9
	**Hybrid**	85.03	99.40	79.00	0.88	1	450

AC- Amino acid composition, DC dipeptide composition, PSSM position specific scoring matrix, ACC accuracy, SN- Sensitivity, SP- specificity, MCC- Mathews correlation coefficient,C: tradeoff value, γ- gamma factor (a parameter in RBF kernel).

SAK - Staphylokinase, SK - Streptokinase, tPA - tissue plasminogen activators, UK - Urokinase.

### Hybrid (AC + DC) SVM modules

A hybrid prediction method combining amino acid composition (AC) and dipeptide composition (DC) was also attempted to solve the Pg-activator protein prediction problem [21]. Using the hybrid approach, accuracy, sensitivity, specificity and MCC were 85.63%, 97.71%, 77.06%, and 0.85 respectively (Table 1). A comparison of the performance of various Pg-activators classifiers is shown in Table 2. For the hybrid model, the accuracy was 87.29%, 89.33%, 83.63%, and 84.48% for SAK, SK, tPA and UK respectively. In general the results of the hybrid compared to the individual models show an increase in sensitivity at the cost of a decrease in specificity, providing marginal improvement overall.

### PSSM profile based SVM modules

In order to improve the performance of SVM models, position specific score matrix (PSSM) profile based prediction models for Pg-activators were also developed and achieved a maximum accuracy of 87.61% with 95.77%, 81.81% sensitivity, specificity and 0.86 of MCC (Table 1). The parameters were subsequently optimized for predicting Pg-activators subclass and achieved a maximum accuracy of 93.98%, 97.10%, 97.73%, 92.06% with MCC of 0.92, 0.97, 0.98, 0.90 for SAK, SK, tPA and UK respectively. In general all models performed comparatively well as estimated by accuracy and MCC measures, including the simple AC method (Table 2).

### Prediction result analysis

SVM- prediction results obtained with optimized kernel parameters on known Pg-act peptides are shown in Additional file 1 a,b,c for SK, Additional file 2-a,b,c for SAK, Additional file 3-a,b,c for UK and Additional file 4 for tPA of amino acid(AC), dipeptide(DC) and PSSM profiles composition respectively. It can be seen that a large number of activators are predicted over a threshold of 0.9 and all are predicted above threshold 0.5.

### Prediction scoring graphs and confusion matrix analysis

**Figure 3:**
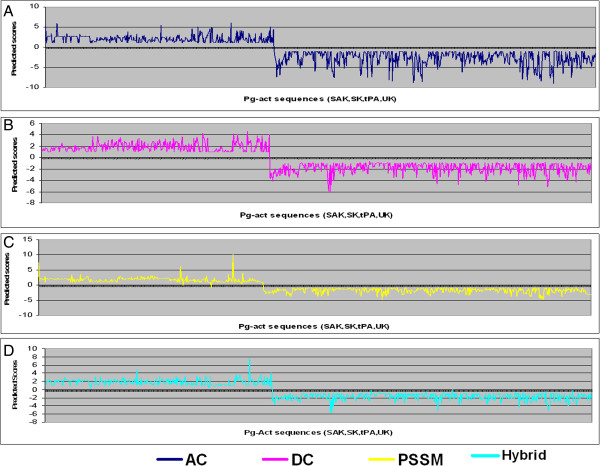
**Prediction scores graph of all Pg-activators Vs non Pg-activators.** The prediction scores graph was generated by the best model which implemented in our online server. **A)**. AC - method, **B)**. DC - method **C)**. PSSM - method and **D)**. Hybrid - method (combination of AC and DC methods). (X-axis is indexed on Pg-activators and Y-axis is the prediction score).

**Figure 4:**
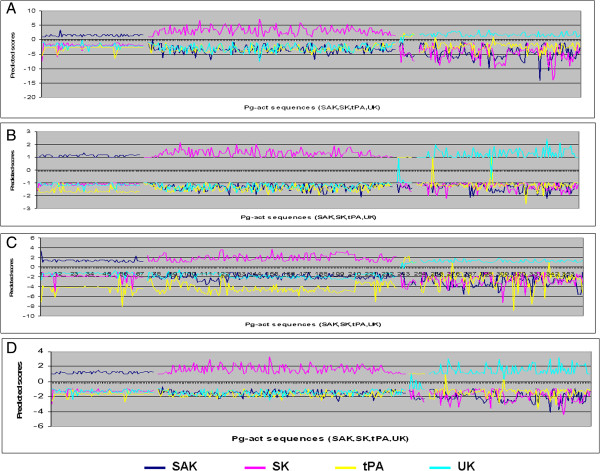
**Prediction scores graph for Sub-class Pg-activators.** The best model of SAK, SK, tPA and UK was used to generate the prediction scores graph which used in online-server. **A)**. AC - Method of SAK, SK, tPA and UK Pg-activators, **B)**. DC - method of SAK, SK, tPA and UK Pg-activators **C)**. PSSM - method of SAK, SK, tPA and UK Pg-activators and **D)**. Hybrid – method (combination of AC and DC methods) of SAK, SK, tPA and UK Pg-activators. (X-axis is indexed on Pg-activators of (SAK, SK, tPA and UK in respective order) and Y- axis is the score of the prediction).

The performance of SVM modules was also checked by examination of the confusion matrix and scoring graphs. The scoring graph represents the score of the prediction for all individual sequences tested (indexed on the horizontal axes), showing how the score of sequences in the positive set (left side of the graph) is separated from the negative set (right side) by a threshold (typically set to zero) that can be used to classify positive and negative predictions. However, not all positive or negative sequences may be correctly classified, leading to false negative and false positive prediction outcomes. To represent this aspect of the performance, the confusion matrix summarizes the outcomes of the prediction (Table 3) [22]. The analysis shows that only two Pg-activator sequences are negatively predicted in the PSSM model while amino, dipeptide and Hybrid models predicted all sequences in the test set correctly (Figures 3 and 4). In subfamilies classification, all subclasses were predicted correctly. The only misclassification was that tPA sequences were also often positively predicted by UK models and, conversely, UK sequences were sometimes positively predicted by tPA classifiers. This observation suggests some overlap between UK and tPA sequence composition. More interestingly, there is no confusion in the correct recognition of positive sequences of their corresponding models. The false positive rate (FPR) estimated on the negative dataset was 0.17, 0.25 and 0.18 in AC, DC and PSSM profile respectively.

**Table 3:** Confusion matrix of all Pg-act proteins (SAK, SK, tPA and UK) against the SVM best models (implemented in the Pg-act server)

	***Total***	***SAK***				***SK***				***tPA***				***UK***			
**Methods**		**AC**	**DC**	**PSSM**	**Hybrid**	**AC**	**DC**	**PSSM**	**Hybrid**	**AC**	**DC**	**PSSM**	**Hybrid**	**AC**	**DC**	**PSSM**	**Hybrid**
**SAK**	69	69	69	69	69	0	0	0	0	0	0	0	0	0	0	0	0
**SK**	167	0	0	0	0	167	167	167	167	0	0	0	0	0	0	0	0
**tPA**	11	0	0	0	0	0	0	0	0	5	11	11	10	6	0	0	1
**UK**	109	0	0	0	0	0	0	0	0	0	2	2	2	109	107	107	107

### ROC-Plot (receiver operating characteristic plot)

**Figure 5:**
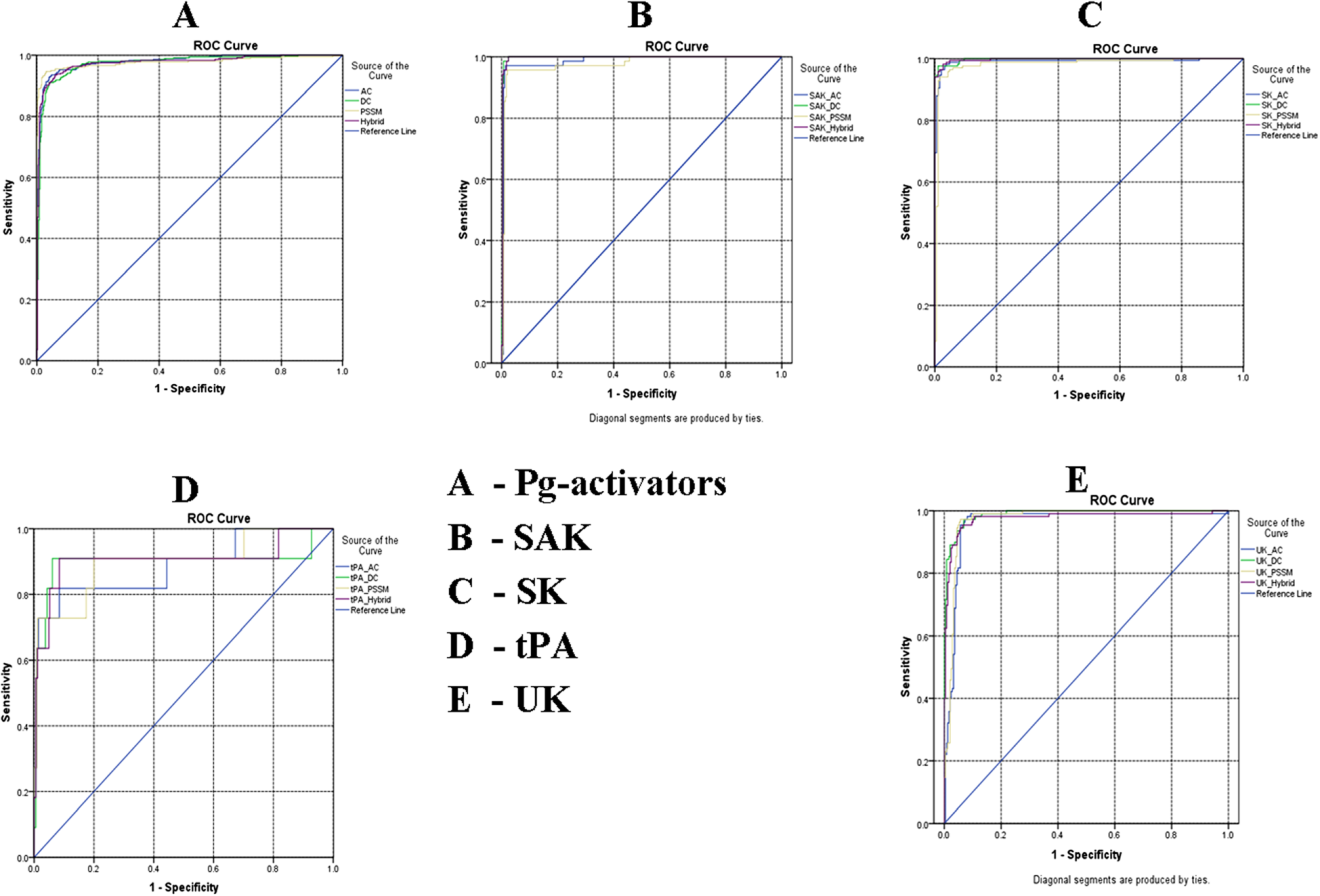
**ROC- plot: The performance of SVM models for all Pg-activators developed using all methods. A)**. Pg-activators in all methods (AC, DC, PSSM and Hybrid). **B)**. SAK Pg-activators in AC, DC, PSSM and Hybrid methods. **C)**. SK Pg-activators in AC, DC, PSSM and Hybrid methods. **D)**. tPA Pg-activators in AC, DC, PSSM and Hybrid methods. **E)**. UK Pg-activators in AC, DC, PSSM and Hybrid methods.

In order to confirm the performance of our models, we plotted the sensitivity versus 1-specificity chart, also known as receiver operating curve (ROC) [23]. It is a two-dimensional depiction of classifier performance [24,25]. The area under the curve for Pg-activators for amino, dipeptide, PSSM and Hybrid was 0.980, 0.974, 0.977, 0.975 respectively. In the classification of Pg activators, we achieved AUC of 0.986 0.996, 0.975, 0.996 for SAK, 0.991, 0.998, 0.982, 0.998 for SK, 0.886, 0.899, 0.898, 0.906 for tPA, 0.962, 0.989, 0.973, 0.977 for UK with the amino, dipeptide, PSSM and Hybrid methods respectively (Figure 5).

### Prediction of new Pg-activators

In order to assess further if the classifier is able to predict Pg-activators not included in the original dataset used for training, new pg-activator sequences were retrieved from the latest versions of the Uniprot/Swissprot database, and tested in the pg-activator prediction server. A total of 207 new sequences previously unseen by the SVM algorithm were obtained (Additional file 5). All these sequences were correctly predicted as positive by all pg-activator models (AC, DC, PSSM and Hybrid models). Interestingly, this included vampire bat saliva and Snake Venom pg-activators, further supporting the efficacy of positive prediction on novel sequences.

### Web server

A freely accessible web server has been developed using simple html format, cgi-Perl programs and the optimized SVM algorithm for prediction of Pg-activators sequences. Firstly, our program predicts whether the input sequence is a pg-activators or not. Secondly, if the answer is positive, the sequence is assigned to the most likely Pg-activators subclass. The input sequence can be any format and our program allow user to make prediction with different prediction accuracy thresholds.

## Discussion

At present, a large number of methods have been developed to predict the function of proteins. Most of these methods have been developed for protein structure prediction, subcellular localization and functional classification of proteins [26-28]. In this study, we have made a systematic attempt to predict Pg-activator proteins and their sub-classes through SVM. Streptokinase is a 414 amino-acid peptide secreted by many β-hemolytic streptococcal species. Streptokinase is composed of three domains (residues 1–150), β (residues 151–287), and γ (residues 288–414). Unlike urokinase (UK) and tPA that cleave the plasminogen bond Arg561-Val562 to generate plasmin, Streptokinase (SK) lacks intrinsic protease activity. Instead, SK forms a stoichiometric complex with Pg and coverts it to an activator complex. Staphylokinase is a single domain protein of 136 amino acids. It also like SK, forms a stoichiometric complex with plasminogen and the SK + Pg complex then activates plasminogen.

All Pg-activators can activate Pg but they have different mechanisms of action and specificity. Here, through a comprehensive approach we used amino acid, dipetide composition, PSSM profile and Hybrid methods to predict the putative Pg activation abilities of novel protein sequences. However, the approach is purely based on sequence composition and this does not explain any mechanistic details of Pg activation. So identifying and predicting the amino acid sequence which are related to differential mechanism and different specificity may need more rigorous computational as well as experimental methods. However, the results show that good predictions can be obtained from sequence composition alone, suggesting that certain features of sequence allow the useful discrimination of Pg-activators. However, SVM does not easily permit the direct identification of the feature having the most influence in any details, and this aspect will need to be explored further.

The PgActPred server can predict Pg-activators as well as in their SAK, SK, tPA or UK sub-class with high accuracy, sensitivity, specificity and MCC. In general, dipeptide (DC) and PSSM models predict better than the simple amino acid composition models (AC).

## Conclusion

In this study, for the first time, a method has been developed for the prediction of Pg-activators and their sub-classification in subfamilies such as Streptokinase (SK), Staphylokinase (SAK), tissue plasminogen activators (tPA) and Urokinase (UK) from their amino acid sequence alone. It has been observed that all methods implemented are comparable predictors, including the simple amino acid composition model. A confusion matrix has been produced to cross check the predicted results, showing all prokaryotic pg-activators (SK, SAK) are correctly classified while few eukaryotic Pg-activators are misclassified. Remarkably, Cysteine (C) residues are absent in all prokaryotic Pg-activators. Tremendous amount of lysine (K) is present in prokaryotic Pg-activators, so this residue may play important contributions to indirect plasminogen activators. Pg-activators perform a unique function even though they come from different origins and our study and tools facilitate the annotation of pg-activators.

## Methods

### Datasets

The data of all Pg-activators were extracted from Uniprot/SWISSPROT database. The data set was created by removing sequences annotated as “fragments”, “isoforms”, “potentials”, “similarity”, or “probables”. This dataset was further processed with the CD-hit program to remove sequences with more than 90% similarity with any other in the dataset to avoid redundancy and incorporation of variants [29]. The final dataset contains 356 Pg-activators (positive dataset). The negative dataset consisted of 501 non Pg-activator sequences randomly selected out of 19534 regulatory proteins. The Pg-activators containing 69 Staphylokinase (SAK), 167 streptokinase (SK), 11 tissue plasminogen activator (tPA), and 109 of urokinase (UK) sub-family sequences respectively. The negative sequence set was selected by the general keyword query for “regulatory proteins” in Uniprot/Swisprot (regulatory proteins from various species).

### Amino acid and dipeptide composition

The amino acid composition is the fraction of each amino acid in a protein. The SVM_light requires the encoding of data into vectors. So the fraction of all 20 natural amino acids was calculated using the following equation:

(1) $$\begin{array}{l} \text{Fraction}\;\mathrm{of}\;\text{amino}\;\text{acid}\;\left(i\right)\\ \;=\frac{\text{Total}\;\text{number}\;\mathrm{of}\;\text{amino}\;\text{acid}\;\left(i\right)}{\text{Total}\;\text{number}\;\mathrm{of}\;\text{amino}\;\text{acids}\;\mathrm{in}\;\text{protein}} \end{array}$$

Where (*i*) can be any amino acid.

Similarly, dipeptide composition has been calculated by a vector having a fixed length of 400 (20 × 20) dimensions. The fraction of each dipeptide composition was calculated by the following equation:

(2) $$\begin{array}{l} \text{Fraction}\;\mathrm{of}\;\text{dipeptide}\;\left(i\right)\\ \;=\frac{\text{Total}\;\text{number}\;\mathrm{of}\;\text{dipep}\;\left(i\right)}{\text{Total}\;\text{number}\;\mathrm{of}\;\text{all}\;\text{possible}\;\text{dipeptides}} \end{array}$$

### PSSM profile

PSSM profile was generated using the GPSR package against nr (non redundant) blast database. In gpsr package we have used seq2pssm_imp, pssm_n2, pssm_comp and col2svm programs for PSI-BLAST searches against the nr database using different iterations with a cutoff e-value 0.001, and to normalize the PSSM profile and generate the SVM_light input format (i.e. as a composition vector of 400) [30]. Finally, the SVM models were generated using different parameters, optimized and, the best model used in the prediction server.

### Evaluation and performance

A five-fold cross validation technique has been used to evaluate performance. Firstly we used Pg-activators as positive dataset and non Pg-activators as negative dataset. Both positive and negative datasets were randomly divided into five equal sets. To run SVM, four sets were used for training and the remaining set for testing. This process was repeated five times, so that each sub-set was used once for testing. This has been applied to all methods i.e. amino acid, dipeptide, PSSM and Hybrid predictions of Pg-activators. The final performance was calculated by average the results of testing on all five sets. The sensitivity, specificity, accuracy and Mathew correlation coefficient (MCC), were adopted for performance evaluation of the classifiers. These measures were calculated with the following standard formulas;

(3) $$\text{Accuracy}=\frac{\mathrm{TP}+\mathrm{TN}}{\mathrm{TP}+\mathrm{TN}+\mathrm{FP}+\mathrm{FN}}$$

(4) $$\text{Sensitivity}=\frac{\mathrm{TP}}{\mathrm{TP}+\mathrm{FN}}$$

(5) $$\text{Specificity}=\frac{\mathrm{TN}}{\mathrm{TP}+\mathrm{FP}}$$

(6) $$\text{MCC}=\frac{\mathrm{TP}\times \mathrm{TN}-\mathrm{FP}\times \mathrm{FN}}{\sqrt{\left(\mathrm{TP}+\mathrm{FP}\right)\left(\mathrm{TP}+\mathrm{FN}\right)\left(\mathrm{TN}+\mathrm{FP}\right)\left(\mathrm{TN}+\mathrm{FN}\right)}}$$

Where TP, FP, TN and FN represent the number of true positive, false positive, true negative and false negatives respectively.

### Support vector machine

The SVM_light, a highly successful machine learning technique, has been used for the prediction of plasminogen activators. The SVM can use various parameter settings like kernel, linear, polynomial and radial basic functions (RBI). In the prediction studies, we have optimized different parameter for each prediction method. In the first approach all Pg-activators were used as a positive and the non Pg-activators as negative examples. The same approach was also applied for classification in one sub-class of Pg-activators using all other sub-classes sequences as a negative example. In practice, we applied (+)ve label for positive sequence and (-)ve label for negative sequence to run SVM light.

## Competing interests

The authors have declared that they have no competing interests.

## Authors’ contributions

MK carried developing of web server with guidance from CL. MK and CL participated in developing computational methods and performed the statistical analysis. MK, CL and MP conceived the study, and participated in its design and coordination and helped to draft the manuscript. All authors read and approved the final manuscript.

## Supplementary Material

Additional file 1 **The streptokinase (SK) SVM best models predicted all streptokinase (SK) proteins and sorted from Minimum scores to maximum scores corresponding to their protein ID (Uniprot/Swiss prot).** The models of AC, DC and PSSM of 1a, 1b and 1crespectively. The construction of min to max score tables shows to easily to identify the unknown and new SK proteins according to their predicted scores. Click here for file

Additional file 2 **The all staphylokinase (SAK) proteins predicted by all best models of AC, DC and PSSM, the scores sorted by the minimum to maximum according to their protein ID (Uniprot/Swiss prot) as 2a, 2b and 2c respectively.** Using this table, the unknown and similar SAK proteins easily can identify by using the predicted scores. Click here for file

Additional file 3 **The urokinase (UK) proteins of all predicted by their best models of AC, DC and PSSM, the predicted scores sorted as minimum to the maximum according to their protein ID (Uniprot/Swiss prot) as 3a, 3b and 3c respectively.** The unknown and similar UK proteins can easily identify using the predicted scores. Click here for file

Additional file 4 **The tissue plasminogen activators (tPA) models such as AC, DC, and PSSM predicted all tPA proteins, the scores sorted from minimum to maximum displayed according to their Uniprot/Swiss prot protein ID.** The new and unknown tPA proteins can easily identify the similar proteins according to their predicted scores. Click here for file

Additional file 5 The list of new and unknown of Pg-activator sequences, which was not in our dataset, predicted as Pg-activators in our all models (AC, DC, PSSM and Hybrid). Click here for file
