# Supplementary material for: Support vector machine (SVM) based multiclass prediction with basic statistical analysis of plasminogen activators
Source: BMC Res Notes. 2014 Jan 27;7:63. doi: 10.1186/1756-0500-7-63 (PMC3924408; doi:10.1186/1756-0500-7-63)
Supplement: Additional file 1 — The streptokinase (SK) SVM best models predicted all streptokinase (SK) proteins and sorted from Minimum scores to maximum scores corresponding to their protein ID (Uniprot/Swiss prot). The models of AC, DC and PSSM of 1a, 1b and 1crespectively. The construction of min to max score tables shows to easily to identify the unknown and new SK proteins according to their predicted scores. [file 1756-0500-7-63-S1.doc]

**Additional file 1**

The streptokinase(SK) SVM best models predicted all streptokinase (SK)proteins and sorted from Minimum scores to maximum scores corresponding to theirprotein ID (Uniprot / Swiss prot). The models of AC, DC and PSSM of 1a, 1b and 1crespectively. The construction of min to max scores tables shows to easily toidentify the unknown and new SK proteins according to their predicted scores.

***Additional file 1a***

| ***SK_AC*** | ***AC*** | ***SK_AC*** | ***AC*** | ***SK_AC*** | ***AC*** | ***SK_AC*** | ***AC*** | ***SK_AC*** | ***AC*** |
| --- | --- | --- | --- | --- | --- | --- | --- | --- | --- |
| Q8KQN3_STREQ | 0.9966674 | Q7X0V9_STRPY | 1.9258673 | Q7X0Q7_STRPY | 2.5949 | Q7X0T7_STRPY | 3.2933739 | Q7X0W2_STREQ | 4.272594 |
| LRPR_STREQ | 0.9975591 | Q1J4K8_STRPF | 1.9382817 | Q54688_STRPY | 2.6077 | Q7X0R9_STRPY | 3.3549505 | Q7X0X3_STREQ | 4.27605 |
| DTD_STREQ | 0.9981315 | Q7X0U6_STRPY | 1.9476697 | Q6UK57_STREQ | 2.616 | Q9ZFE3_STREQ | 3.3967884 | Q7X0P9_STRPY | 4.302399 |
| Q8KQN4_STREQ | 0.9988986 | Q7X0S9_STRPY | 1.9524768 | Q9ZFE2_STREQ | 2.6186 | Q54681_STRPY | 3.4831082 | Q7X0W1_STREQ | 4.322234 |
| Q55278_9STRE | 1.0004833 | Q7X0Q2_STRPY | 1.9616364 | Q7X0R8_STRPY | 2.6979 | Q7B2W7_STRUB | 3.5288632 | Q93U16_STRUB | 4.337398 |
| Q2YI45_STRAG | 1.0021066 | Q7X0Q5_STRPY | 1.9709851 | Q7X0S5_STRPY | 2.7046 | B9DW72_STRU0 | 3.5288632 | Q7X0X6_STREQ | 4.353103 |
| DEXB_STREQ | 1.0031251 | Q7X0Q6_STRPY | 1.9955976 | Q7X0Q8_STRPY | 2.7359 | Q7X0V0_STRPY | 3.5358621 | Q7X0T9_STRPY | 4.435406 |
| Q2YI46_STRAG | 1.0089701 | Q7X0X7_STRPY | 2.0716794 | Q7X0R6_STRPY | 2.7561 | Q7X0R7_STRPY | 3.5773414 | Q7X0V4_STRPY | 4.458265 |
| RELA_STREQ | 1.0093549 | Q7X0V3_STRPY | 2.0768411 | Q7X0V2_STRPY | 2.8281 | B5SVI5_STRPY | 3.6638217 | Q7X0Q3_STRPY | 4.466726 |
| B5SVH6_STRPY | 1.262189 | Q57391_STRPY | 2.0835546 | B5SVI0_STRPY | 2.8369 | Q54687_STRPY | 3.6798947 | Q7X0U8_STRPY | 4.687071 |
| Q55279_STRSG | 1.2933851 | B5XIM5_STRPZ | 2.0835546 | STRP_STRS1 | 2.8517 | Q7X0Y3_STRPY | 3.6857732 | Q7X0X4_STREQ | 4.754443 |
| Q54692_STRPY | 1.3335265 | Q7X0S7_STRPY | 2.0989875 | Q54682_STRPY | 2.8752 | Q7M115_STRSP | 3.68955 | Q7X0U9_STRPY | 4.837173 |
| C0M9F5_STRE4 | 1.3486833 | Q7X0T5_STRPY | 2.0989875 | Q7X0R1_STRPY | 2.9039 | Q7X0P6_STRPY | 3.7068112 | Q7X0P8_STRPY | 4.969398 |
| B4U591_STREM | 1.4103063 | Q54685_STRPY | 2.1335837 | Q8NZA6_STRP8 | 2.9146 | Q54686_STRPY | 3.7143902 | Q7X0W9_STREQ | 5.044057 |
| STRQ_STRPY | 1.4187936 | Q7X0T0_STRPY | 2.1589075 | Q7X0X8_STRPY | 2.9174 | Q7X0V7_STRPY | 3.7570151 | Q7X0U3_STRPY | 5.077724 |
| Q8KQN2_STREQ | 1.4238349 | Q7X0Q4_STRPY | 2.1630445 | Q7X0S3_STRPY | 2.9515 | B5SVI6_STRPY | 3.8015783 | Q7X0W0_STREQ | 5.096196 |
| B5SVH2_STRPY | 1.4423808 | Q7X0V1_STRPY | 2.1915749 | C5WED5_STRDG | 2.9718 | B5SVI4_STRPY | 3.8026303 | Q7X0W7_STREQ | 5.137859 |
| C0MF92_STRS7 | 1.4481526 | Q7X0S2_STRPY | 2.2569144 | Q7X0Y7_STRPY | 2.973 | Q7X0Q0_STRPY | 3.8062073 | Q7X0U7_STRPY | 5.195052 |
| Q7X0R3_STRPY | 1.4714347 | B8Y6H8_9STRE | 2.2629376 | Q5X9T6_STRP6 | 2.973 | Q7X0V6_STRPY | 3.863814 | Q7X0S6_STRPY | 5.224271 |
| Q1JJU9_STRPC | 1.4928435 | Q7X0X9_STRPY | 2.278163 | Q7X0U1_STRPY | 2.9933 | Q6LCU0_STRPY | 3.8705794 | Q7X0W8_STREQ | 5.282165 |
| Q1J9Q2_STRPB | 1.4928435 | Q7X0T2_STRPY | 2.2972472 | Q54691_STRPY | 2.999 | Q7X0S1_STRPY | 3.8841488 | Q7X0X0_STREQ | 5.323432 |
| Q48R78_STRPM | 1.6615506 | Q54684_STRPY | 2.3110564 | Q7X0V8_STRPY | 3.055 | Q7X0R0_STRPY | 3.9519061 | B5SVJ2_STRPY | 5.392076 |
| Q7X0R5_STRPY | 1.6662611 | Q54683_STRPY | 2.4080205 | Q7X0Y8_STRPY | 3.0716 | Q7X0V5_STRPY | 3.976545 | Q7X0W6_STREQ | 5.41911 |
| Q54693_STRPY | 1.6823563 | STRP_STREQ | 2.4353802 | Q8K5R8_STRP3 | 3.116 | Q7X0Y2_STRPY | 3.9979584 | Q54695_STRPY | 5.480803 |
| A8YQE3_STRPY | 1.683911 | C5WGK4_STRDG | 2.4603978 | Q53284_STREQ | 3.1161 | Q7X0T3_STRPY | 4.0012921 | Q7X0W4_STREQ | 5.49643 |
| Q7X0Y6_STRPY | 1.7210715 | Q7X0U5_STRPY | 2.4708468 | Q7X0Y1_STRPY | 3.1189 | A0FJ59_9STRE | 4.0519028 | Q7X0W5_STREQ | 5.547533 |
| Q7X0P7_STRPY | 1.734296 | Q7X0T1_STRPY | 2.4789795 | Q7X0Y0_STRPY | 3.1787 | Q7X0X2_STREQ | 4.1076454 | Q7X0U4_STRPY | 5.657507 |
| Q7X0R4_STRPY | 1.7406601 | Q54680_STRPY | 2.4822556 | STRP_STRP1 | 3.1932 | Q7X0R2_STRPY | 4.1310624 | B5SVI9_STRPY | 5.692753 |
| Q54689_STRPY | 1.7444474 | Q7X0U2_STRPY | 2.5119988 | Q53ZX6_STRPY | 3.1932 | Q7X0T6_STRPY | 4.1692449 | Q7X0X1_STREQ | 5.858696 |
| B5ATV6_STRPY | 1.8377666 | Q7X0Y4_STRPY | 2.5538267 | B5SVH1_STRPY | 3.2109 | Q7WS87_STREQ | 4.1829633 | Q7X0S0_STRPY | 6.713889 |
| Q54690_STRPY | 1.8586992 | Q1JEU1_STRPD | 2.5538267 | O87869_STRUB | 3.2118 | Q7X0T4_STRPY | 4.1993439 | Q9R4S1_STREQ | 7.071802 |
| Q7X0T8_STRPY | 1.8774318 | Q7X0Q1_STRPY | 2.5720647 | Q7X0U0_STRPY | 3.2169 | Q7X0W3_STREQ | 4.2401694 |  |  |
| Q54694_STRPY | 1.9161147 | Q7X0Q9_STRPY | 2.5852315 | Q7X0Y5_STRPY | 3.2364 | Q7X0S4_STRPY | 4.2512038 |  |  |
| Q7X0S8_STRPY | 1.9224007 | Q54086_STREQ | 2.5855106 | Q7B2W0_STRUB | 3.2864 | Q7X0X5_STREQ | 4.260734 |  |  |

Additional file 1b

| ***SK_DC*** | ***DC*** | ***SK_DC*** | ***DC*** | ***SK_DC*** | ***DC*** | ***SK_DC*** | ***DC*** | ***SK_DC*** | ***DC*** |
| --- | --- | --- | --- | --- | --- | --- | --- | --- | --- |
| Q8KQN3_STREQ | 0.99882843 | Q6UK57_STREQ | 1.0543342 | Q7X0S5_STRPY | 1.204628 | Q7X0X5_STREQ | 1.3816491 | Q7X0S8_STRPY | 1.549374 |
| Q8KQN4_STREQ | 1.0000007 | Q7X0Y8_STRPY | 1.0549731 | Q7X0X6_STREQ | 1.209153 | Q7X0R0_STRPY | 1.3847748 | Q7X0T2_STRPY | 1.559875 |
| Q9ZFE2_STREQ | 1.0002493 | Q7X0Y6_STRPY | 1.0553287 | Q7X0V9_STRPY | 1.218731 | Q7X0Q4_STRPY | 1.3937019 | Q7X0Q1_STRPY | 1.561216 |
| Q9ZFE3_STREQ | 1.0005046 | C0MF92_STRS7 | 1.0555384 | Q6LCU0_STRPY | 1.221872 | Q7X0R6_STRPY | 1.3950513 | Q54695_STRPY | 1.566998 |
| Q7B2W0_STRUB | 1.0009824 | C5WED5_STRDG | 1.056143 | Q7X0Y5_STRPY | 1.22774 | Q7X0Q6_STRPY | 1.3962195 | Q7X0T6_STRPY | 1.571433 |
| Q54086_STREQ | 1.0012616 | Q1JJU9_STRPC | 1.0565493 | Q7X0Q5_STRPY | 1.228288 | Q7X0R9_STRPY | 1.3965184 | Q7X0S0_STRPY | 1.577096 |
| DTD_STREQ | 1.0015432 | Q1J9Q2_STRPB | 1.0565493 | Q7X0U5_STRPY | 1.239402 | Q54684_STRPY | 1.4017189 | Q7X0Q0_STRPY | 1.590795 |
| RELA_STREQ | 1.0017007 | Q7X0Y0_STRPY | 1.0580877 | Q7X0X7_STRPY | 1.239902 | Q7X0V2_STRPY | 1.4042069 | Q7X0V8_STRPY | 1.621769 |
| Q55278_9STRE | 1.0035438 | STRP_STRS1 | 1.0602486 | Q54688_STRPY | 1.245598 | B5SVH2_STRPY | 1.405264 | B5SVI9_STRPY | 1.624218 |
| Q7X0R8_STRPY | 1.0035487 | Q8K5R8_STRP3 | 1.0711115 | Q7X0Y3_STRPY | 1.248593 | Q7X0R2_STRPY | 1.409591 | Q7X0T4_STRPY | 1.642843 |
| B8Y6H8_9STRE | 1.0039348 | Q7X0Y4_STRPY | 1.078207 | Q7X0T0_STRPY | 1.260881 | B5SVH1_STRPY | 1.4241513 | Q54686_STRPY | 1.645485 |
| B5SVI0_STRPY | 1.0040308 | Q1JEU1_STRPD | 1.078207 | Q7X0T7_STRPY | 1.269187 | Q54693_STRPY | 1.4271076 | B5SVJ2_STRPY | 1.647877 |
| STRP_STRP1 | 1.0042204 | Q54683_STRPY | 1.0817486 | Q7X0W4_STREQ | 1.273785 | Q54690_STRPY | 1.4301721 | Q7X0V0_STRPY | 1.654737 |
| Q53ZX6_STRPY | 1.0042204 | Q7X0U0_STRPY | 1.0863885 | Q7X0W6_STREQ | 1.284679 | Q7X0Q9_STRPY | 1.4302815 | Q7X0P8_STRPY | 1.675769 |
| B5ATV6_STRPY | 1.0057718 | Q54680_STRPY | 1.1151627 | B5SVI4_STRPY | 1.288088 | Q7X0U1_STRPY | 1.4400066 | Q7X0U7_STRPY | 1.677182 |
| DEXB_STREQ | 1.0064485 | Q53284_STREQ | 1.1166116 | Q7X0X3_STREQ | 1.289004 | Q7X0T9_STRPY | 1.4425858 | Q7X0U3_STRPY | 1.683538 |
| STRQ_STRPY | 1.0065506 | Q7X0P7_STRPY | 1.1188925 | A0FJ59_9STRE | 1.298353 | Q7X0U2_STRPY | 1.4434163 | Q7X0U4_STRPY | 1.694744 |
| O87869_STRUB | 1.0072433 | Q7X0Q7_STRPY | 1.1332991 | Q7X0S7_STRPY | 1.298621 | Q7X0S9_STRPY | 1.4522724 | Q7X0Q3_STRPY | 1.696448 |
| STRP_STREQ | 1.0073416 | Q57391_STRPY | 1.1375861 | Q7X0W5_STREQ | 1.30039 | Q7X0X1_STREQ | 1.4711543 | B5SVI5_STRPY | 1.709327 |
| Q2YI45_STRAG | 1.0089804 | B5XIM5_STRPZ | 1.1375861 | Q54682_STRPY | 1.312563 | Q7X0U6_STRPY | 1.4722885 | Q7M115_STRSP | 1.727841 |
| LRPR_STREQ | 1.0089915 | Q54694_STRPY | 1.1390788 | Q7X0X0_STREQ | 1.313682 | Q54681_STRPY | 1.4757922 | Q7X0R7_STRPY | 1.762397 |
| Q48R78_STRPM | 1.0091488 | Q8NZA6_STRP8 | 1.1454184 | Q7X0W3_STREQ | 1.316916 | Q54692_STRPY | 1.4789771 | Q7X0U9_STRPY | 1.768997 |
| Q1J4K8_STRPF | 1.0094425 | Q7B2W7_STRUB | 1.1462773 | Q7X0X2_STREQ | 1.321331 | Q7X0R4_STRPY | 1.4881133 | Q7X0P6_STRPY | 1.788719 |
| C0M9F5_STRE4 | 1.0114839 | B9DW72_STRU0 | 1.1462773 | Q7X0V6_STRPY | 1.327755 | Q7X0T1_STRPY | 1.4916298 | Q7X0P9_STRPY | 1.79817 |
| Q2YI46_STRAG | 1.0148619 | Q7X0Q8_STRPY | 1.1480594 | Q7X0V5_STRPY | 1.328365 | Q7X0V1_STRPY | 1.4939432 | Q7X0V7_STRPY | 1.800368 |
| B4U591_STREM | 1.0150263 | B5SVH6_STRPY | 1.1534484 | Q54691_STRPY | 1.331774 | Q7X0W2_STREQ | 1.4954424 | Q7X0S6_STRPY | 1.809584 |
| C5WGK4_STRDG | 1.0165087 | Q7X0X9_STRPY | 1.1536856 | Q7X0W0_STREQ | 1.352576 | Q7X0Q2_STRPY | 1.5001221 | Q7X0T3_STRPY | 1.819649 |
| A8YQE3_STRPY | 1.0174369 | Q54687_STRPY | 1.1623894 | Q7X0S3_STRPY | 1.357763 | Q7X0T8_STRPY | 1.501748 | B5SVI6_STRPY | 1.92562 |
| Q8KQN2_STREQ | 1.0180778 | Q54689_STRPY | 1.1744498 | Q7X0R1_STRPY | 1.361388 | Q7X0S2_STRPY | 1.5123091 | Q7X0U8_STRPY | 2.006208 |
| Q7X0X8_STRPY | 1.0212302 | Q93U16_STRUB | 1.1754475 | Q7WS87_STREQ | 1.365209 | Q7X0V3_STRPY | 1.5161305 | Q9R4S1_STREQ | 2.032003 |
| Q7X0Y2_STRPY | 1.048728 | Q7X0X4_STREQ | 1.1812461 | Q7X0W8_STREQ | 1.378123 | Q7X0R3_STRPY | 1.5247529 | Q7X0V4_STRPY | 2.178161 |
| Q7X0Y1_STRPY | 1.0492459 | Q55279_STRSG | 1.1813843 | Q7X0W1_STREQ | 1.378875 | Q7X0W7_STREQ | 1.5311992 |  |  |
| Q7X0Y7_STRPY | 1.0513585 | Q7X0R5_STRPY | 1.1846635 | Q54685_STRPY | 1.380105 | Q7X0W9_STREQ | 1.532821 |  |  |
| Q5X9T6_STRP6 | 1.0513585 | Q7X0T5_STRPY | 1.2006763 | Q7X0S4_STRPY | 1.380988 | Q7X0S1_STRPY | 1.5340879 |  |  |

Additional file 1c

| ***SK_PSSM*** | ***PSSM*** | ***SK_PSSM*** | ***PSSM*** | ***SK_PSSM*** | ***PSSM*** | ***SK_PSSM*** | ***PSSM*** | ***SK_PSSM*** | ***PSSM*** |
| --- | --- | --- | --- | --- | --- | --- | --- | --- | --- |
| Q9ZFE2_STREQ | 0.607113 | Q54691_STRPY | 1.2649114 | Q7X0R0_STRPY | 1.577138 | STRQ_STRPY | 1.9512437 | Q7X0W1_STREQ | 2.641732 |
| Q2YI45_STRAG | 0.751911 | Q7X0X7_STRPY | 1.2664348 | Q7X0Y6_STRPY | 1.578845 | Q48R78_STRPM | 1.9585848 | Q7X0S3_STRPY | 2.659406 |
| C0M9F5_STRE4 | 0.999626 | Q7X0U4_STRPY | 1.2691151 | Q7X0X9_STRPY | 1.582071 | Q7B2W7_STRUB | 1.9884577 | Q7X0V9_STRPY | 2.65971 |
| Q1J4K8_STRPF | 0.999935 | Q7X0Q4_STRPY | 1.2798587 | B5SVI0_STRPY | 1.584372 | Q54690_STRPY | 2.0087904 | Q7X0T2_STRPY | 2.665469 |
| Q1JEU1_STRPD | 0.999935 | B4U591_STREM | 1.2854343 | STRP_STRS1 | 1.589246 | Q7X0Y1_STRPY | 2.0307726 | Q7X0X4_STREQ | 2.67501 |
| Q7X0Y4_STRPY | 0.999956 | Q7X0R3_STRPY | 1.2863856 | Q54683_STRPY | 1.593003 | Q7X0T5_STRPY | 2.0487813 | Q7X0X2_STREQ | 2.692714 |
| Q7X0Y7_STRPY | 0.999977 | Q7X0S6_STRPY | 1.2908466 | Q7X0Q0_STRPY | 1.604672 | Q7X0Q9_STRPY | 2.0866224 | Q7X0Q2_STRPY | 2.730291 |
| DTD_STREQ | 0.999988 | Q7X0T0_STRPY | 1.2930912 | Q7X0Q8_STRPY | 1.66334 | Q7X0S0_STRPY | 2.135392 | Q7X0U6_STRPY | 2.830084 |
| Q7X0Y8_STRPY | 1 | Q7X0X5_STREQ | 1.3127717 | Q8KQN2_STREQ | 1.666209 | Q54680_STRPY | 2.2342371 | Q7X0V2_STRPY | 2.894872 |
| DEXB_STREQ | 1.000418 | Q93U16_STRUB | 1.3127717 | STRP_STRP1 | 1.666632 | Q7X0U8_STRPY | 2.2972828 | Q7X0W7_STREQ | 2.942039 |
| Q8K5R8_STRP3 | 1.000432 | Q54688_STRPY | 1.3128849 | Q53284_STREQ | 1.666632 | Q8KQN3_STREQ | 2.3202011 | Q55278_9STRE | 2.946702 |
| C5WGK4_STRDG | 1.00068 | Q7X0U9_STRPY | 1.3207243 | Q7X0X6_STREQ | 1.67286 | Q7X0V3_STRPY | 2.3415214 | Q7X0P7_STRPY | 2.954204 |
| Q1JJU9_STRPC | 1.024375 | Q7X0Q3_STRPY | 1.3305056 | Q9ZFE3_STREQ | 1.68483 | Q7X0V4_STRPY | 2.361904 | Q7X0W8_STREQ | 2.956494 |
| C0MF92_STRS7 | 1.025594 | Q7X0Q5_STRPY | 1.3428352 | Q7X0V0_STRPY | 1.688268 | B5SVI4_STRPY | 2.3841282 | Q7X0U5_STRPY | 2.978282 |
| RELA_STREQ | 1.066262 | Q7X0U0_STRPY | 1.3487528 | Q54086_STREQ | 1.689312 | Q7X0T1_STRPY | 2.394749 | Q54695_STRPY | 3.000712 |
| Q2YI46_STRAG | 1.100793 | Q7X0U3_STRPY | 1.3615442 | Q54682_STRPY | 1.694968 | Q7WS87_STREQ | 2.4379526 | B5SVJ2_STRPY | 3.04184 |
| B5SVH6_STRPY | 1.121786 | Q54692_STRPY | 1.3747725 | Q7X0T8_STRPY | 1.699738 | B5SVI5_STRPY | 2.4441294 | Q7X0W5_STREQ | 3.046143 |
| Q7X0P6_STRPY | 1.12484 | Q1J9Q2_STRPB | 1.3918649 | Q6LCU0_STRPY | 1.722548 | Q7X0R6_STRPY | 2.4551319 | Q7X0W6_STREQ | 3.046395 |
| B5ATV6_STRPY | 1.12519 | Q7X0Y2_STRPY | 1.3918649 | Q7X0X8_STRPY | 1.722785 | Q54686_STRPY | 2.4723909 | Q7X0V8_STRPY | 3.056892 |
| Q7X0R2_STRPY | 1.125991 | Q7X0S1_STRPY | 1.4159009 | LRPR_STREQ | 1.723597 | Q7X0P9_STRPY | 2.4854014 | Q7X0U1_STRPY | 3.0606 |
| A8YQE3_STRPY | 1.126479 | Q54693_STRPY | 1.4282255 | Q7X0S2_STRPY | 1.735994 | Q7X0V5_STRPY | 2.490457 | Q7X0U7_STRPY | 3.060625 |
| B5SVH2_STRPY | 1.147133 | Q53ZX6_STRPY | 1.4307189 | Q6UK57_STREQ | 1.74665 | A0FJ59_9STRE | 2.4967277 | B5SVI9_STRPY | 3.096654 |
| Q7X0T6_STRPY | 1.180376 | Q7X0S9_STRPY | 1.4480173 | C5WED5_STRDG | 1.764894 | B5SVI6_STRPY | 2.5050863 | Q7X0W4_STREQ | 3.134468 |
| Q7X0V7_STRPY | 1.180923 | Q54684_STRPY | 1.451475 | B9DW72_STRU0 | 1.782584 | Q7X0T4_STRPY | 2.5098241 | Q7X0W3_STREQ | 3.13767 |
| Q7X0Q1_STRPY | 1.186155 | STRP_STREQ | 1.4788589 | Q7B2W0_STRUB | 1.782584 | Q7X0X0_STREQ | 2.5247868 | Q7X0U2_STRPY | 3.158336 |
| O87869_STRUB | 1.189101 | Q54689_STRPY | 1.5044296 | Q9R4S1_STREQ | 1.792689 | Q5X9T6_STRP6 | 2.5257977 | Q7X0S5_STRPY | 3.184769 |
| Q7X0S7_STRPY | 1.189903 | Q7X0R5_STRPY | 1.5409143 | Q7X0Y3_STRPY | 1.803893 | Q54685_STRPY | 2.5376721 | Q7X0W2_STREQ | 3.215937 |
| B8Y6H8_9STRE | 1.216059 | Q57391_STRPY | 1.5451315 | Q7X0V6_STRPY | 1.814444 | Q7X0R1_STRPY | 2.5443555 | Q8KQN4_STREQ | 3.217669 |
| Q7X0S8_STRPY | 1.2160832 | Q7X0Y5_STRPY | 1.5451315 | Q7X0T9_STRPY | 1.843757 | Q7X0P8_STRPY | 2.5592444 | Q54694_STRPY | 3.2316 |
| B5XIM5_STRPZ | 1.2277381 | Q8NZA6_STRP8 | 1.5451869 | Q54681_STRPY | 1.848833 | Q7M115_STRSP | 2.5752273 | Q7X0W9_STREQ | 3.351741 |
| Q55279_STRSG | 1.2277381 | Q7X0Q6_STRPY | 1.5670594 | B5SVH1_STRPY | 1.861439 | Q7X0X3_STREQ | 2.576829 | Q7X0R9_STRPY | 3.68578 |
| Q7X0T3_STRPY | 1.2365091 | Q7X0Q7_STRPY | 1.5692026 | Q7X0R8_STRPY | 1.912639 | Q7X0X1_STREQ | 2.5847106 |  |  |
| Q7X0V1_STRPY | 1.2412874 | Q7X0S4_STRPY | 1.5719107 | Q54687_STRPY | 1.933843 | Q7X0T7_STRPY | 2.5966897 |  |  |
| Q7X0R4_STRPY | 1.2427302 | Q7X0R7_STRPY | 1.5730901 | Q7X0Y0_STRPY | 1.948494 | Q7X0W0_STREQ | 2.6244498 |  |  |
