# Supplementary material for: Support vector machine (SVM) based multiclass prediction with basic statistical analysis of plasminogen activators
Source: BMC Res Notes. 2014 Jan 27;7:63. doi: 10.1186/1756-0500-7-63 (PMC3924408; doi:10.1186/1756-0500-7-63)
Supplement: Additional file 2 — The all staphylokinase (SAK) proteins predicted by all best models of AC, DC and PSSM, the scores sorted by the minimum to maximum according to their protein ID (Uniprot/Swiss prot) as 2a, 2b and 2c respectively. Using this table, the unknown and similar SAK proteins easily can identify by using the predicted scores. [file 1756-0500-7-63-S2.doc]

**Additional file 2**

The all staphylokinase (SAK) proteins predicted by all best models of AC, DCand PSSM, the scores sorted by the minimum to maximum according to their proteinID(Uniprot / Swiss prot) as 2a, 2b and 2c respectively. Using this table, the unknown andsimilar SAK proteins easily can identify by using the predicted scores.

Additional file 2a

| ***SAK_AC*** | ***AC*** | ***SAK_AC*** | ***AC*** | ***SAK_AC*** | ***AC*** | ***SAK_AC*** | ***AC*** | ***SAK_AC*** | ***AC*** |
| --- | --- | --- | --- | --- | --- | --- | --- | --- | --- |
| A8HZI8_STAA | 1.000038 | C8MWH3_STAA | 1.0796111 | SAK_STAAM | 1.33716 | SAK_STAAR | 1.8622865 | C8L8C1_STAA | 1.8622865 |
| SAK_BPP42 | 1.000152 | C8LGA4_STAA | 1.0796111 | SAK_STAAS | 1.33716 | A0EX12_9CAU | 1.8622865 | C2G5Y2_STAA | 1.8622865 |
| Q53721_STAA | 1.000227 | Q9LC43_STAA | 1.1961772 | SAK_STAAU | 1.33716 | C8ATH2_STAA | 1.8622865 | C8A633_STAA | 1.8622865 |
| A2I7K2_STAA | 1.001898 | C3VIQ5_STAA | 1.2116931 | D1QCQ5_STAA | 1.33716 | C8MCD7_STAA | 1.8622865 | D2G2U9_STAA | 1.8622865 |
| Q38085_9VIR | 1.001905 | A7TWR8_9VIR | 1.3063967 | C8KPU7_STAA | 1.33716 | D2F8L4_STAA | 1.8622865 | C8AL66_STAA | 1.8622865 |
| ATKA2_STAAU | 1.002059 | C5Q4M8_STAA | 1.3063967 | C5QL00_STAA | 1.33716 | D2GT46_STAA | 1.8622865 | A8Z2V9_STAA | 1.8622865 |
| ATKC2_STAAU | 1.00393 | D2FEI4_STAA | 1.3063967 | C5MYU9_STAA | 1.33716 | Q9LC46_STAA | 1.8622865 | A5IUB3_STAA | 1.8622865 |
| Q9LC49_STAA | 1.005516 | D2UNU0_STAA | 1.3063967 | C8M4E2_STAA | 1.33716 | C8ADH3_STAA | 1.8622865 | A6U349_STAA | 1.8622865 |
| TAGA_STAAU | 1.006921 | D1R2E8_STAA | 1.3063967 | A7X486_STAA | 1.33716 | C8MZ51_STAA | 1.8622865 | Q2FFF5_STAA | 1.8622865 |
| SECY_ECOLI | 1.008288 | Q9AM04_STAA | 1.3063967 | SAK_BPPHD | 1.605583 | C2K6A0_STAA | 1.8622865 | A6QIH0_STAA | 1.8622865 |
| SAK_STAAN | 1.079611 | C8LXE2_STAA | 1.3063967 | Q2FWV3_STAA | 1.605583 | D2GB60_STAA | 1.8622865 | Q38086_9VIR | 1.8746803 |
| D1QIX1_STAA | 1.079611 | D2FNK8_STAA | 1.3063967 | O33929_STAA | 1.759924 | D2UTN0_STAA | 1.8622865 | C8MM53_STAA | 1.9903731 |
| C8L3Q7_STAA | 1.0796111 | D1GPD4_STAA | 1.3063967 | Q9LC47_STAA | 1.793319 | C7ZYI7_STAA | 1.8622865 | TAGH_STAAU | 3.4758593 |
| D3EXZ3_STAA | 1.0796111 | SAK_STAAW | 1.3172733 | Q4ZCZ1_9CAU | 1.814852 | D2FWU4_STAA | 1.8622865 |  |  |

| ***SAK_DC*** | ***DC*** | ***SAK_DC*** | ***DC*** | ***SAK_DC*** | ***DC*** | ***SAK_DC*** | ***DC*** | ***SAK_DC*** | ***DC*** |
| --- | --- | --- | --- | --- | --- | --- | --- | --- | --- |
| Q38086_9VIR | 0.99973664 | ATKC2_STAAU | 1.0102397 | C8LXE2_STAA | 1.143702 | C2K6A0_STAA | 1.1665655 | A6QIH0_STAA | 1.1665655 |
| TAGH_STAAU | 0.99979206 | A2I7K2_STAA | 1.0111618 | D2FNK8_STAA | 1.143702 | D2GB60_STAA | 1.1665655 | SAK_STAAM | 1.1671012 |
| ATKA2_STAAU | 1.0014078 | Q9LC49_STAA | 1.0119624 | D1GPD4_STAA | 1.143702 | D2UTN0_STAA | 1.1665655 | SAK_STAAS | 1.1671012 |
| Q9LC47_STAA | 1.0022859 | Q4ZCZ1_9CAU | 1.0128866 | C3VIQ5_STAA | 1.157452 | C7ZYI7_STAA | 1.1665655 | SAK_STAAU | 1.1671012 |
| SAK_STAAN | 1.0035589 | TAGA_STAAU | 1.017028 | SAK_STAAW | 1.158507 | D2FWU4_STAA | 1.1665655 | D1QCQ5_STAA | 1.1671012 |
| D1QIX1_STAA | 1.0035589 | SAK_BPP42 | 1.0933934 | SAK_STAAR | 1.166566 | C8L8C1_STAA | 1.1665655 | C8KPU7_STAA | 1.1671012 |
| C8L3Q7_STAA | 1.0035589 | A8HZI8_STAA | 1.1311703 | A0EX12_9CAU | 1.166566 | C2G5Y2_STAA | 1.1665655 | C5QL00_STAA | 1.1671012 |
| D3EXZ3_STAA | 1.0035589 | C8MM53_STAA | 1.1404207 | C8ATH2_STAA | 1.166566 | C8A633_STAA | 1.1665655 | C5MYU9_STAA | 1.1671012 |
| C8MWH3_STAA | 1.0035589 | A7TWR8_9VIR | 1.1437023 | C8MCD7_STAA | 1.166566 | D2G2U9_STAA | 1.1665655 | C8M4E2_STAA | 1.1671012 |
| C8LGA4_STAA | 1.0035589 | C5Q4M8_STAA | 1.1437023 | D2F8L4_STAA | 1.166566 | C8AL66_STAA | 1.1665655 | A7X486_STAA | 1.1671012 |
| SECY_ECOLI | 1.0066829 | D2FEI4_STAA | 1.1437023 | D2GT46_STAA | 1.166566 | A8Z2V9_STAA | 1.1665655 | SAK_BPPHD | 1.1840931 |
| Q9LC43_STAA | 1.0069769 | D2UNU0_STAA | 1.1437023 | Q9LC46_STAA | 1.166566 | A5IUB3_STAA | 1.1665655 | Q2FWV3_STAA | 1.1840931 |
| Q38085_9VIR | 1.0079681 | D1R2E8_STAA | 1.1437023 | C8ADH3_STAA | 1.166566 | A6U349_STAA | 1.1665655 | O33929_STAA | 1.3404181 |
| Q53721_STAA | 1.0096258 | Q9AM04_STAA | 1.1437023 | C8MZ51_STAA | 1.166566 | Q2FFF5_STAA | 1.1665655 |  |  |

Additional file 2b

Additional file 2c

| ***SAK_PSSM*** | ***PSSM*** | ***SAK_PSSM*** | ***PSSM*** | ***SAK_PSSM*** | ***PSSM*** | ***SAK_PSSM*** | ***PSSM*** | ***SAK_PSSM*** | ***PSSM*** |
| --- | --- | --- | --- | --- | --- | --- | --- | --- | --- |
| A2I7K2_STAA | 0.99902295 | D1QIX1_STAA | 1.0480123 | A7TWR8_9VIR | 1.244368 | C8MM53_STAA | 1.4819171 | C8MCD7_STAA | 1.5104019 |
| Q4ZCZ1_9CAU | 0.99934627 | D3EXZ3_STAA | 1.0480123 | C5Q4M8_STAA | 1.244368 | A5IUB3_STAA | 1.5104019 | C8MZ51_STAA | 1.5104019 |
| ATKC2_STAAU | 0.99945212 | SAK_BPP42 | 1.0814267 | C8LXE2_STAA | 1.244368 | A6QIH0_STAA | 1.5104019 | D2F8L4_STAA | 1.5104019 |
| Q9LC47_STAA | 0.99952113 | A8HZI8_STAA | 1.1822084 | D1GPD4_STAA | 1.244368 | SAK_STAAR | 1.5104019 | D2FWU4_STAA | 1.5104019 |
| TAGA_STAAU | 0.99981374 | SAK_STAAM | 1.1997716 | D1R2E8_STAA | 1.244368 | A6U349_STAA | 1.5104019 | D2G2U9_STAA | 1.5104019 |
| Q38085_9VIR | 1.0005105 | SAK_STAAS | 1.1997716 | D2FEI4_STAA | 1.244368 | A8Z2V9_STAA | 1.5104019 | D2GB60_STAA | 1.5104019 |
| Q53721_STAA | 1.0006549 | SAK_STAAU | 1.1997716 | D2FNK8_STAA | 1.244368 | C2G5Y2_STAA | 1.5104019 | D2GT46_STAA | 1.5104019 |
| Q9LC49_STAA | 1.0007415 | A7X486_STAA | 1.1997716 | D2UNU0_STAA | 1.244368 | C2K6A0_STAA | 1.5104019 | D2UTN0_STAA | 1.5104019 |
| Q9LC43_STAA | 1.0008425 | C5MYU9_STAA | 1.1997716 | Q9AM04_STAA | 1.244368 | C7ZYI7_STAA | 1.5104019 | Q2FFF5_STAA | 1.5104019 |
| SECY_ECOLI | 1.0009052 | C5QL00_STAA | 1.1997716 | C3VIQ5_STAA | 1.266325 | C8A633_STAA | 1.5104019 | Q9LC46_STAA | 1.5104019 |
| SAK_STAAN | 1.0480123 | C8KPU7_STAA | 1.1997716 | Q2FWV3_STAA | 1.369821 | C8ADH3_STAA | 1.5104019 | Q38086_9VIR | 1.6596221 |
| C8L3Q7_STAA | 1.0480123 | C8M4E2_STAA | 1.1997716 | SAK_BPPHD | 1.369821 | C8AL66_STAA | 1.5104019 | TAGH_STAAU | 2.4370972 |
| C8LGA4_STAA | 1.0480123 | D1QCQ5_STAA | 1.1997716 | O33929_STAA | 1.387467 | C8ATH2_STAA | 1.5104019 | A0EX12_9CAU | 3.2047915 |
| C8MWH3_STAA | 1.0480123 | SAK_STAAW | 1.2101201 | ATKA2_STAAU | 1.397974 | C8L8C1_STAA | 1.5104019 |  |  |
