# Supplementary material for: Support vector machine (SVM) based multiclass prediction with basic statistical analysis of plasminogen activators
Source: BMC Res Notes. 2014 Jan 27;7:63. doi: 10.1186/1756-0500-7-63 (PMC3924408; doi:10.1186/1756-0500-7-63)
Supplement: Additional file 3 — The urokinase (UK) proteins of all predicted by their best models of AC, DC and PSSM, the predicted scores sorted as minimum to the maximum according to their protein ID (Uniprot/Swiss prot) as 3a, 3b and 3c respectively. The unknown and similar UK proteins can easily identify using the predicted scores. [file 1756-0500-7-63-S3.doc]

**Additional file 3**

The urokinase (UK) proteins of all predicted by their best models ofAC, DC and PSSM, the predicted scores sorted as minimum to the maximum accordicgto their protein ID (Uniprot / Swiss prot) as 3a, 3b and 3c respectively. The unknown andsimilar UK proteins can easily identify using the predicted scores.

Additional file 3a

| ***UK_AC*** | ***AC*** | ***UK_AC*** | ***AC*** | ***UK_AC*** | ***AC*** | ***UK_AC*** | ***AC*** | ***UK_AC*** | ***AC*** |
| --- | --- | --- | --- | --- | --- | --- | --- | --- | --- |
| Q95M89_HORSE | 0.99686895 | Q9DRY4_COWPX | 1.0047714 | Q5SWW8_HUMAN | 1.59048 | PDGFD_MOUSE | 1.814696 | LYPD3_MOUSE | 2.316611 |
| B0WH75_CULQU | 0.99735614 | PHCA_ARTFS | 1.1341628 | D3GE88_DANRE | 1.640234 | UROK_PIG | 1.8229591 | UPAR_AOTTR | 2.324401 |
| PAI2_MOUSE | 0.99804096 | HABP2_HUMAN | 1.2311321 | PDGFD_RAT | 1.646744 | HABP2_MOUSE | 1.8236789 | NID1_HUMAN | 2.327671 |
| Q6GY32_HUMAN | 0.99828718 | SPB12_MOUSE | 1.2451081 | TMPS6_HUMAN | 1.646763 | MRC2_HUMAN | 1.8305534 | PAI1_MUSVI | 2.595089 |
| PDGFD_RABIT | 0.99877723 | HABP2_BOVIN | 1.2471867 | UPAR_PANTR | 1.655599 | GDN_RAT | 1.8379891 | PA2I_NAJKA | 2.601201 |
| Q9XT70_RABIT | 0.99887214 | PDGFD_HUMAN | 1.25855 | UROK_PONAB | 1.667307 | B4DNJ4_HUMAN | 1.8421327 | WFKN1_MOUSE | 2.658227 |
| Q6SLL2_CANFA | 0.9989209 | Q9TVA8_BOVIN | 1.3613459 | Q3KR76_RAT | 1.66844 | B0W3W5_CULQU | 1.9651627 | PAI1_HUMAN | 2.678419 |
| Q90675_CHICK | 0.99954268 | SACA4_HUMAN | 1.3672319 | UPAR_MACFA | 1.677692 | UROK_PAPCY | 1.9703462 | LYPD3_HUMAN | 2.705063 |
| UROK_CHICK | 0.99989689 | VTNC_HUMAN | 1.3759945 | GDN_HUMAN | 1.683324 | HABP2_RAT | 1.9747496 | WFKN1_RAT | 2.719681 |
| Q66VU5_PICAN | 0.99991009 | C6GBR3_SHEEP | 1.3832948 | Q9R119_CRIGR | 1.691589 | HGFA_HUMAN | 1.9876471 | PAI1_PIG | 2.746349 |
| Q96SE8_HUMAN | 1.0001912 | UPAR_MOUSE | 1.392431 | Q5PY49_HUMAN | 1.701965 | UPAR_RAT | 2.0186566 | C6GBR4_SHEEP | 2.837531 |
| Q206M5_LAMVI | 1.0003085 | Q545X5_MOUSE | 1.392431 | UROK_RAT | 1.709709 | POLS3_MOUSE | 2.0370393 | Q0X0F2_HUMAN | 2.877976 |
| SPB12_HUMAN | 1.0007538 | UROK_RABIT | 1.4322398 | UPAR_CERAE | 1.719785 | CENPR_HUMAN | 2.0531187 | Q0X0F0_RAT | 2.968063 |
| IVBI1_PSETT | 1.0008314 | SPA33_BOVIN | 1.4369073 | UROK_BOVIN | 1.724704 | WFKN1_HUMAN | 2.1258525 | Q0X0F1_MOUSE | 2.978942 |
| NFKB1_HUMAN | 1.0010925 | O97587_RABIT | 1.4473046 | UROK_HUMAN | 1.727874 | IPSP_HUMAN | 2.1388831 | PAI1_MOUSE | 3.091982 |
| Q59GZ8_HUMAN | 1.0011763 | LRP2_HUMAN | 1.4573984 | MRC2_MOUSE | 1.733846 | UPAR_BOVIN | 2.1420916 | PAI1_BOVIN | 3.12264 |
| SPA31_BOVIN | 1.0015439 | UPAR_HUMAN | 1.4750784 | Q8MKB1_RABIT | 1.735672 | TMPS6_MOUSE | 2.1489934 | PAI1_RAT | 3.142544 |
| Q9UEJ5_HUMAN | 1.0020416 | PDGFD_PONAB | 1.4865901 | B2R7F2_HUMAN | 1.736709 | PAI2_HUMAN | 2.1493476 | O55006_RAT | 3.160166 |
| HNRPC_HUMAN | 1.0022592 | POLS3_HUMAN | 1.4927589 | MRC2_RAT | 1.737293 | Q9TVA9_BOVIN | 2.2128848 | TF65_HUMAN | 3.426154 |
| B0X3R2_CULQU | 1.0031143 | UROK_MOUSE | 1.5225269 | Q9N124_HORSE | 1.748151 | GDN_MOUSE | 2.2141787 | REL_HUMAN | 3.428575 |
| PHCB_ARTFS | 1.0037715 | Q0VBA8_MOUSE | 1.5225269 | A5HC32_RABIT | 1.772868 | ST14_HUMAN | 2.2999165 |  |  |
| PAI2_RAT | 1.0039249 | B0X1R8_CULQU | 1.528277 | Q8SPU1_MACMU | 1.799423 | PA2J_NAJKA | 2.3067508 |  |  |

Additional file 3b

| ***UK_DC*** | ***DC*** | ***UK_DC*** | ***DC*** | ***UK_DC*** | ***DC*** | ***UK_DC*** | ***DC*** | ***UK_DC*** | ***DC*** |
| --- | --- | --- | --- | --- | --- | --- | --- | --- | --- |
| IVBI1_PSETT | 0.9529842 | SPB12_HUMAN | 1.0046215 | D3GE88_DANRE | 1.015842 | SPA33_BOVIN | 1.3329495 | UPAR_MOUSE | 1.646107 |
| C6GBR3_SHEEP | 0.998766 | LRP2_HUMAN | 1.004641 | PAI2_RAT | 1.033373 | TF65_HUMAN | 1.3342449 | Q545X5_MOUSE | 1.646107 |
| IPSP_HUMAN | 0.9993903 | PAI2_MOUSE | 1.0056479 | PAI1_BOVIN | 1.038012 | Q0X0F0_RAT | 1.3484019 | UROK_MOUSE | 1.650407 |
| PAI1_MUSVI | 0.9996337 | B0X3R2_CULQU | 1.0056923 | HABP2_RAT | 1.055911 | Q6SLL2_CANFA | 1.3654804 | Q0VBA8_MOUSE | 1.650407 |
| Q6GY32_HUMAN | 0.9996355 | PAI1_PIG | 1.0060908 | UPAR_MACFA | 1.067715 | UROK_PONAB | 1.3785881 | TMPS6_MOUSE | 1.678121 |
| CENPR_HUMAN | 0.9997351 | GDN_MOUSE | 1.0062853 | UPAR_CERAE | 1.068128 | Q206M5_LAMVI | 1.3801376 | Q3KR76_RAT | 1.693401 |
| Q9UEJ5_HUMAN | 0.9998618 | PDGFD_RABIT | 1.0063852 | PDGFD_PONAB | 1.069308 | Q5PY49_HUMAN | 1.3820633 | O55006_RAT | 1.700688 |
| UROK_CHICK | 1.0000061 | Q9N124_HORSE | 1.0067436 | UPAR_PANTR | 1.07311 | MRC2_RAT | 1.4032524 | UROK_RAT | 1.727033 |
| Q9TVA8_BOVIN | 1.0000954 | PAI1_MOUSE | 1.0068123 | B0X1R8_CULQU | 1.099919 | UROK_HUMAN | 1.4154846 | PA2I_NAJKA | 1.729185 |
| Q95M89_HORSE | 1.0004548 | HABP2_HUMAN | 1.0073076 | Q96SE8_HUMAN | 1.118621 | B2R7F2_HUMAN | 1.4211021 | LYPD3_MOUSE | 1.733509 |
| PHCB_ARTFS | 1.0012292 | SPA31_BOVIN | 1.0088549 | O97587_RABIT | 1.120785 | SACA4_HUMAN | 1.4319393 | WFKN1_HUMAN | 1.750193 |
| Q8SPU1_MACMU | 1.0019413 | Q66VU5_PICAN | 1.0090115 | NID1_HUMAN | 1.151728 | HGFA_HUMAN | 1.4448241 | B0WH75_CULQU | 1.770786 |
| TMPS6_HUMAN | 1.0023075 | B0W3W5_CULQU | 1.0091709 | Q59GZ8_HUMAN | 1.161811 | MRC2_MOUSE | 1.4533571 | POLS3_HUMAN | 1.782887 |
| PDGFD_HUMAN | 1.0024282 | REL_HUMAN | 1.0092164 | PAI1_HUMAN | 1.167764 | WFKN1_RAT | 1.4693595 | Q0X0F2_HUMAN | 1.790094 |
| SPB12_MOUSE | 1.0027353 | GDN_RAT | 1.0099801 | Q0X0F1_MOUSE | 1.225733 | PAI2_HUMAN | 1.4703734 | UPAR_BOVIN | 1.842879 |
| Q90675_CHICK | 1.0028881 | HNRPC_HUMAN | 1.0107237 | PDGFD_RAT | 1.228921 | PDGFD_MOUSE | 1.4926214 | Q9TVA9_BOVIN | 1.897201 |
| NFKB1_HUMAN | 1.0030804 | HABP2_BOVIN | 1.0111176 | ST14_HUMAN | 1.231116 | UROK_RABIT | 1.5088907 | Q9R119_CRIGR | 1.934564 |
| PA2J_NAJKA | 1.0031805 | Q9XT70_RABIT | 1.011635 | Q5SWW8_HUMAN | 1.256407 | WFKN1_MOUSE | 1.5115596 | LYPD3_HUMAN | 2.005141 |
| VTNC_HUMAN | 1.0032419 | Q9DRY4_COWPX | 1.0140948 | UROK_BOVIN | 1.257879 | POLS3_MOUSE | 1.5115795 | C6GBR4_SHEEP | 2.13861 |
| Q8MKB1_RABIT | 1.0039154 | HABP2_MOUSE | 1.0142719 | UPAR_AOTTR | 1.291444 | MRC2_HUMAN | 1.518328 | A5HC32_RABIT | 2.463266 |
| PHCA_ARTFS | 1.0042363 | PAI1_RAT | 1.0146918 | UROK_PIG | 1.294956 | UPAR_RAT | 1.5977907 |  |  |
| GDN_HUMAN | 1.0044921 | UPAR_HUMAN | 1.0153802 | B4DNJ4_HUMAN | 1.306794 | UROK_PAPCY | 1.6224927 |  |  |

Additional file 3c

| ***UK_PSSM*** | ***PSSM*** | ***UK_PSSM*** | ***PSSM*** | ***UK_PSSM*** | ***PSSM*** | ***UK_PSSM*** | ***PSSM*** | ***UK_PSSM*** | ***PSSM*** |
| --- | --- | --- | --- | --- | --- | --- | --- | --- | --- |
| TMPS6_MOUSE | 0.51305039 | HABP2_BOVIN | 1.0006069 | Q59GZ8_HUMAN | 1.301147 | TF65_HUMAN | 1.7042662 | B0W3W5_CULQU | 2.19316 |
| UPAR_BOVIN | 0.99963308 | Q5SWW8_HUMAN | 1.0006331 | PAI2_RAT | 1.310834 | HNRPC_HUMAN | 1.7068153 | HGFA_HUMAN | 2.2698 |
| UROK_BOVIN | 0.99964897 | IVBI1_PSETT | 1.0006862 | PDGFD_MOUSE | 1.315865 | VTNC_HUMAN | 1.7206584 | Q90675_CHICK | 2.372366 |
| GDN_RAT | 0.99971363 | Q95M89_HORSE | 1.0006883 | UPAR_MOUSE | 1.322635 | HABP2_HUMAN | 1.7298558 | NFKB1_HUMAN | 2.431149 |
| UROK_PIG | 0.99975972 | PAI2_MOUSE | 1.0007282 | UPAR_CERAE | 1.331034 | WFKN1_RAT | 1.7756759 | B0X1R8_CULQU | 2.513144 |
| UPAR_AOTTR | 0.99980355 | SPA31_BOVIN | 1.0007914 | C6GBR3_SHEEP | 1.333327 | GDN_HUMAN | 1.798124 | LYPD3_HUMAN | 2.563234 |
| PDGFD_RABIT | 0.9998115 | MRC2_RAT | 1.0009945 | B0X3R2_CULQU | 1.338383 | UPAR_RAT | 1.8019005 | Q0X0F0_RAT | 2.598115 |
| Q8SPU1_MACMU | 0.99988485 | PAI1_RAT | 1.0297519 | PA2I_NAJKA | 1.340772 | REL_HUMAN | 1.8217279 | Q9XT70_RABIT | 2.652362 |
| PDGFD_PONAB | 0.9999101 | Q5PY49_HUMAN | 1.036584 | UROK_PONAB | 1.362462 | C6GBR4_SHEEP | 1.8492109 | UROK_RABIT | 2.733473 |
| Q6SLL2_CANFA | 1.0000042 | UROK_CHICK | 1.0897987 | PAI1_PIG | 1.376646 | Q6GY32_HUMAN | 1.9223179 | Q206M5_LAMVI | 2.797234 |
| CENPR_HUMAN | 1.0000308 | B2R7F2_HUMAN | 1.0933187 | TMPS6_HUMAN | 1.387253 | UPAR_PANTR | 1.9333087 | Q0VBA8_MOUSE | 2.804894 |
| Q8MKB1_RABIT | 1.0001163 | MRC2_HUMAN | 1.141505 | PAI1_BOVIN | 1.393622 | HABP2_MOUSE | 1.9734371 | LRP2_HUMAN | 2.977613 |
| Q0X0F2_HUMAN | 1.0001349 | WFKN1_MOUSE | 1.1415486 | PDGFD_HUMAN | 1.417382 | Q9DRY4_COWPX | 2.010541 | Q9R119_CRIGR | 2.99633 |
| PAI1_MOUSE | 1.0001527 | PAI1_MUSVI | 1.1527809 | Q3KR76_RAT | 1.429814 | UROK_PAPCY | 2.0157856 | POLS3_HUMAN | 3.092816 |
| WFKN1_HUMAN | 1.0001657 | PAI2_HUMAN | 1.1993319 | UPAR_MACFA | 1.429814 | UROK_RAT | 2.0201432 | PHCB_ARTFS | 3.236432 |
| Q96SE8_HUMAN | 1.0002271 | O55006_RAT | 1.2134135 | Q0X0F1_MOUSE | 1.457751 | HABP2_RAT | 2.0392331 | ST14_HUMAN | 3.348051 |
| IPSP_HUMAN | 1.0002548 | MRC2_MOUSE | 1.2155144 | SPB12_MOUSE | 1.514186 | GDN_MOUSE | 2.1050594 | POLS3_MOUSE | 3.526783 |
| PDGFD_RAT | 1.0002777 | LYPD3_MOUSE | 1.2234356 | UPAR_HUMAN | 1.532479 | Q66VU5_PICAN | 2.1218953 | UROK_HUMAN | 3.526783 |
| Q545X5_MOUSE | 1.0002783 | SACA4_HUMAN | 1.2367342 | SPB12_HUMAN | 1.57692 | Q9N124_HORSE | 2.1223547 | O97587_RABIT | 4.3177 |
| SPA33_BOVIN | 1.0002791 | B4DNJ4_HUMAN | 1.2613658 | NID1_HUMAN | 1.614611 | A5HC32_RABIT | 2.1249126 | Q9TVA9_BOVIN | 4.406762 |
| PHCA_ARTFS | 1.0002812 | UROK_MOUSE | 1.2647257 | PA2J_NAJKA | 1.615412 | B0WH75_CULQU | 2.1505969 |  |  |
| D3GE88_DANRE | 1.0003114 | PAI1_HUMAN | 1.266073 | Q9TVA8_BOVIN | 1.689131 | Q9UEJ5_HUMAN | 2.1733583 |  |  |
