# Supplementary material for: Support vector machine (SVM) based multiclass prediction with basic statistical analysis of plasminogen activators
Source: BMC Res Notes. 2014 Jan 27;7:63. doi: 10.1186/1756-0500-7-63 (PMC3924408; doi:10.1186/1756-0500-7-63)
Supplement: Additional file 4 — The tissue plasminogen activators (tPA) models such as AC, DC, and PSSM predicted all tPA proteins, the scores sorted from minimum to maximum displayed according to their Uniprot/Swiss prot protein ID. The new and unknown tPA proteins can easily identify the similar proteins according to their predicted scores. [file 1756-0500-7-63-S4.doc]

**Additional file 4**

The tissue plasminogen activators (tPA) models such as AC, DC, and PSSM predicted all tPA proteins, the scores sorted from minimum to maximum displayedaccording to their Uniprot / Swiss prot protein ID. The newand unknown tPA proteins can easily identify the similar proteins according totheir predicted scores.

| ***tPA_AC*** | ***AC*** | ***tPA_DC*** | ***DC*** | ***tPA_PSSM*** | ***PSSM*** |
| --- | --- | --- | --- | --- | --- |
| IVBI1_PSETT | 0.99696078 | TPA_BOVIN | 0.9958451 | FIBG_HUMAN | 0.999498 |
| LOPAP_LONON | 0.99741958 | LOPAP_LONON | 0.9997321 | LOPAP_LONON | 0.999902 |
| TPA_PONAB | 0.99920823 | TPA_PIG | 1.0013267 | TPA_HUMAN | 0.999986 |
| TPA_MOUSE | 0.99986793 | FIBG_HUMAN | 1.0015284 | TPA_PIG | 0.999996 |
| TPA_PIG | 0.99992972 | Q6P7I9_XENLA | 1.0063931 | TPA_RAT | 1.000171 |
| FIBG_HUMAN | 1.0002692 | IVBI1_PSETT | 1.0067006 | TPA_PONAB | 1.00038 |
| TPA_RAT | 1.0010217 | TPA_HUMAN | 1.0089888 | TPA_MOUSE | 1.000659 |
| TPA_HUMAN | 1.0037124 | Q2PDK2_XENLA | 1.0093147 | IVBI1_PSETT | 1.000701 |
| Q6P7I9_XENLA | 1.5955067 | TPA_RAT | 1.0121437 | Q6P7I9_XENLA | 1.646474 |
| Q2PDK2_XENLA | 2.2405981 | TPA_MOUSE | 1.0135533 | Q2PDK2_XENLA | 1.859161 |
| TPA_BOVIN | 2.293698 | TPA_PONAB | 1.0402515 | TPA_BOVIN | 2.203334 |
